# Improving Documentation of Sleep Assessments in Attention Deficit Hyperactivity Disorder (ADHD) Clinics at Havering Child and Adolescent Mental Health Service

**DOI:** 10.1192/bjo.2025.10376

**Published:** 2025-06-20

**Authors:** Nafiz Imtiaz, Trilok Rana

**Affiliations:** North East London NHS Foundation Trust, London, United Kingdom

## Abstract

**Aims:** Sleep is an essential component of mental well-being. Many patients presenting in the ADHD clinic report sleep issues, which stem from underlying physical or mental health conditions, or as side effects of prescribed medications. These sleep disturbances, if unaddressed may exacerbate existing mental health issues. It can often be challenging to complete a thorough assessment of sleep disturbance in an ADHD clinic in a limited time. Essential components of documentation of sleep assessment were identified from National Institute for Health and Care Excellence (NICE) guidance. Our aim was to achieve 100% documentations containing all essential components of sleep assessment in the ADHD clinic by August 2024.

**Methods:** Measurement: Number of documentations containing all the essential components of sleep assessments were measured.

Initial audit: Initial audit showed only 20% (n=4) records containing all essential components of sleep assessment. Thereafter, three PDSA (plan, do, act, study) cycles were completed as follows:

PDSA Cycle 1: Initial sleep assessment questionnaire was designed and handed over to carer and patient to fill out and uploaded on electronic record system. 50% (n=6) of the documentations contained all the components of sleep assessment.

PDSA Cycle 2: Sleep assessment questionnaire was modified and new questions were added. 70% (n=7) of the documents were found to have all the essential components.

PDSA Cycle 3: Sleep hygiene leaflet introduced. 100% (n=20) of the documentations contained all the essential components.

**Results:** 100% documentations of sleep assessment contained all the essential components. A modified version of the structured sleep assessment questionnaire was designed. A leaflet on sleep hygiene was constructed to improve patient and carer education.

**Conclusion:** Inadequate documentation of sleep disturbance assessment can lead to improper diagnosis, unjudicial prescription of medications, inability to monitor progress or treatment efficacy. Consistent documentation helps in understanding individual sleep patterns in patients with ADHD and helps to enable the clinicians to identify mode of sleep issues which in turn aids in offering adequate management. The use of questionnaire filled out by the carers and patients before the consultation ensured effective assessment and record keeping in a timely fashion and helped as a reference for future comparison to monitor progression and treatment response.

